# Supplementary material for: UniKP: a unified framework for the prediction of enzyme kinetic parameters
Source: Nat Commun. 2023 Dec 11;14:8211. doi: 10.1038/s41467-023-44113-1 (PMC10713628; doi:10.1038/s41467-023-44113-1)
Supplement: Supplementary file 1 — Supplementary Information [file 41467_2023_44113_MOESM1_ESM.pdf]

Supplementary information

UniKP: A unified framework for the prediction of enzyme kinetic parameters

Han Yu[1,2,3,4,†], Huaxiang Deng[1,3,4,†], Jiahui He[1,3,4], Jay D. Keasling[4,5,6,7,8], Xiaozhou Luo[1,2,3,4,*]

[1] Shenzhen Key Laboratory for the Intelligent Microbial Manufacturing of Medicines, Shenzhen Institute of Advanced Technology, Chinese Academy of Sciences, Shenzhen 518055, China

[2] University of Chinese Academy of Sciences, Beijing 100049, China

[3] CAS Key Laboratory of Quantitative Engineering Biology, Shenzhen Institute of Synthetic Biology, Shenzhen Institute of Advanced Technology, Chinese Academy of Sciences, Shenzhen 518055, China

[4] Center for Synthetic Biochemistry, Shenzhen Institute of Synthetic Biology, Shenzhen Institutes of Advanced Technology, Chinese Academy of Sciences, Shenzhen 518055, China

[5] Joint BioEnergy Institute, Emeryville, CA, 94608, USA

[6] Biological Systems and Engineering Division, Lawrence Berkeley National Laboratory, Berkeley, CA, 94720, USA

[7] Department of Chemical and Biomolecular Engineering & Department of Bioengineering, University of California, Berkeley, CA, 94720, USA

[8] Novo Nordisk Foundation Center for Biosustainability, Technical University of Denmark, 2800, Kgs. Lyngby, Denmark

[†] These authors contributed equally to the article.

[*] To whom correspondence should be addressed. Xiaozhou Luo. Email: xz.luo@siat.ac.cn

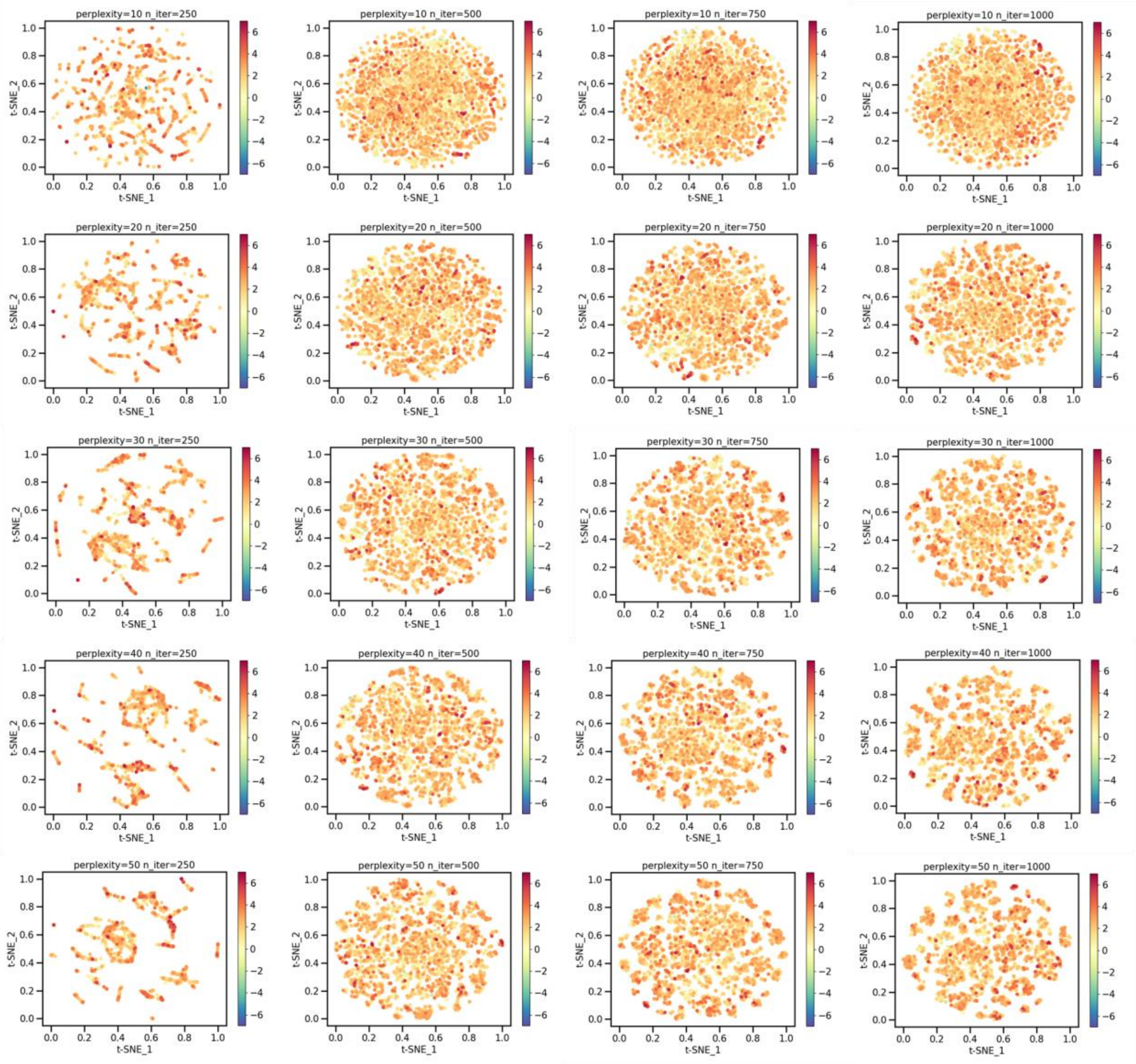

Supplementary Figure 1. Scatter plots showing t-distributed Stochastic Neighbor Embedding (t-SNE) for different perplexity values (10, 20, 30, 40, 50) and iterations (250, 500, 750, 1000) using the DLKcat dataset (N=16,838). The color gradient represents experimentally measured k_{cat} values (logarithm with base 10) of data points, ranging from blue (-7) to red (7). The embedded vectors have been normalized to a range of 0 to 1.

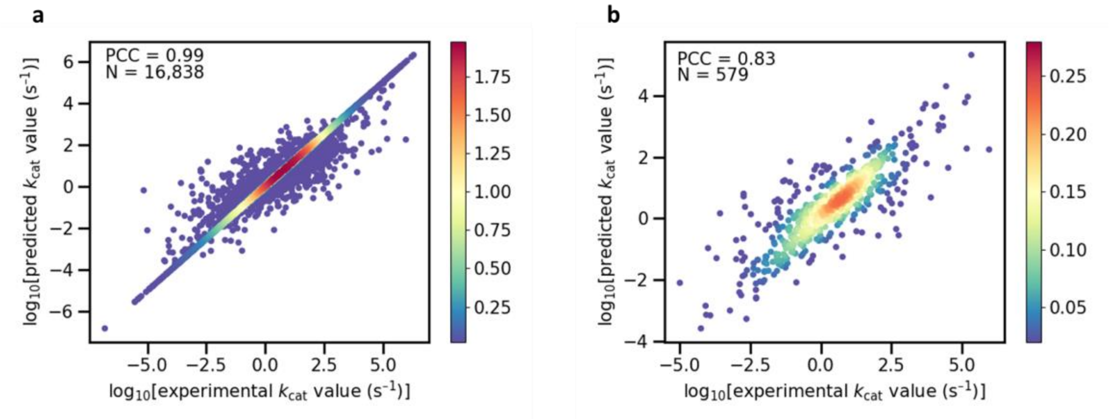

Supplementary Figure 2. a, b) Scatter plot illustrating the Pearson coefficient correlation (PCC) between experimentally measured k_{cat} values and predicted k_{cat} values of UniKP for the entire dataset (**a**) (N=16,838) and strict test set (**b**) (N=579). The color gradient represents the density of data points, ranging from blue (0.02) to red (1.98 or 0.28). Source data are provided as a Source Data file.

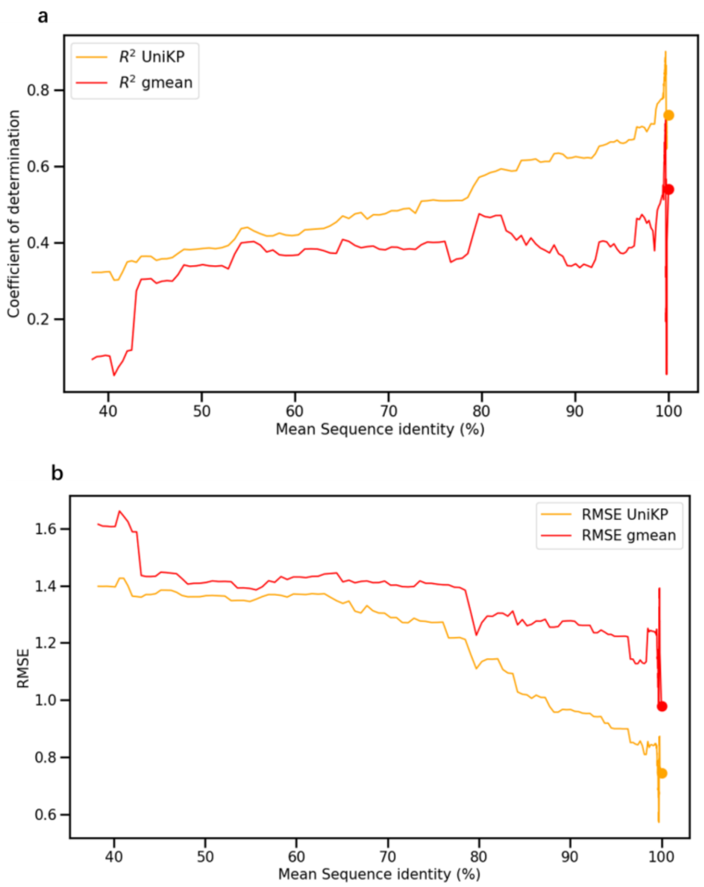

Supplementary Figure 3. a, b) Performance Comparison of UniKP and geometric mean (gmean). The curves are coefficients of variation R^2 (**a**), RMSE (**b**), calculated in sliding windows of size n=100 across sequences in the test set (N=1,679) ordered by the maximal sequence identity between individual test enzymes and all sequences in the training data. Position on the x-axis indicates the mean across the window. Red: UniKP predictions; yellow: geometric mean of k_{cat} values, calculated over the three most similar enzymes in the training set. The two points at the top right are for test datapoints with enzymes already used for training (100% max. sequence identity), these were not included in the sliding windows. Source data are provided as a Source Data file.

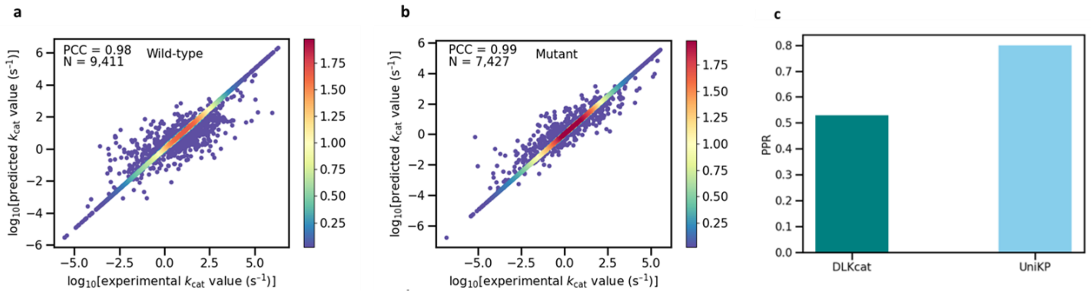

Supplementary Figure 4. a,b) Scatter plot illustrating the Pearson coefficient correlation (PCC) between experimentally measured k_{cat} values and predicted k_{cat} values enzymes of UniKP for the of wild-type (**a**) (N=9,411) and mutated enzymes (**b**) (N=7,427). The color gradient represents the density of data points, ranging from blue (0.02) to red (1.98). **c)** The proportion of predicted accuracy (PPR) values on the test set of DLKcat and UniKP (30 groups). Dark bars represent PPR values of DLKcat and light bars for UniKP. PPR is defined as the proportion of predicted values consistent with the actual values. Source data are provided as a Source Data file.

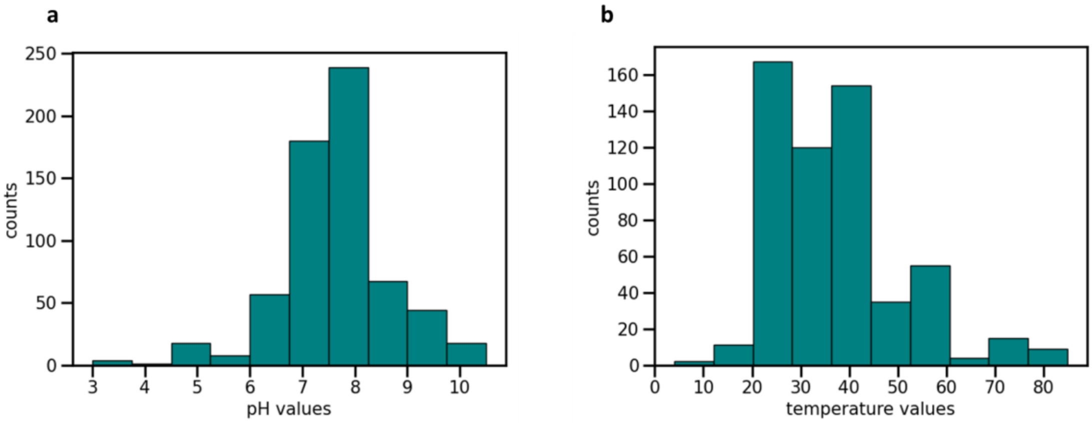

Supplementary Figure 5. a) Histogram of the distribution of pH values in the pH dataset (N=636). **b)** Histogram of the Distribution of temperature values in the temperature dataset (N=572). Source data are provided as a Source Data file.

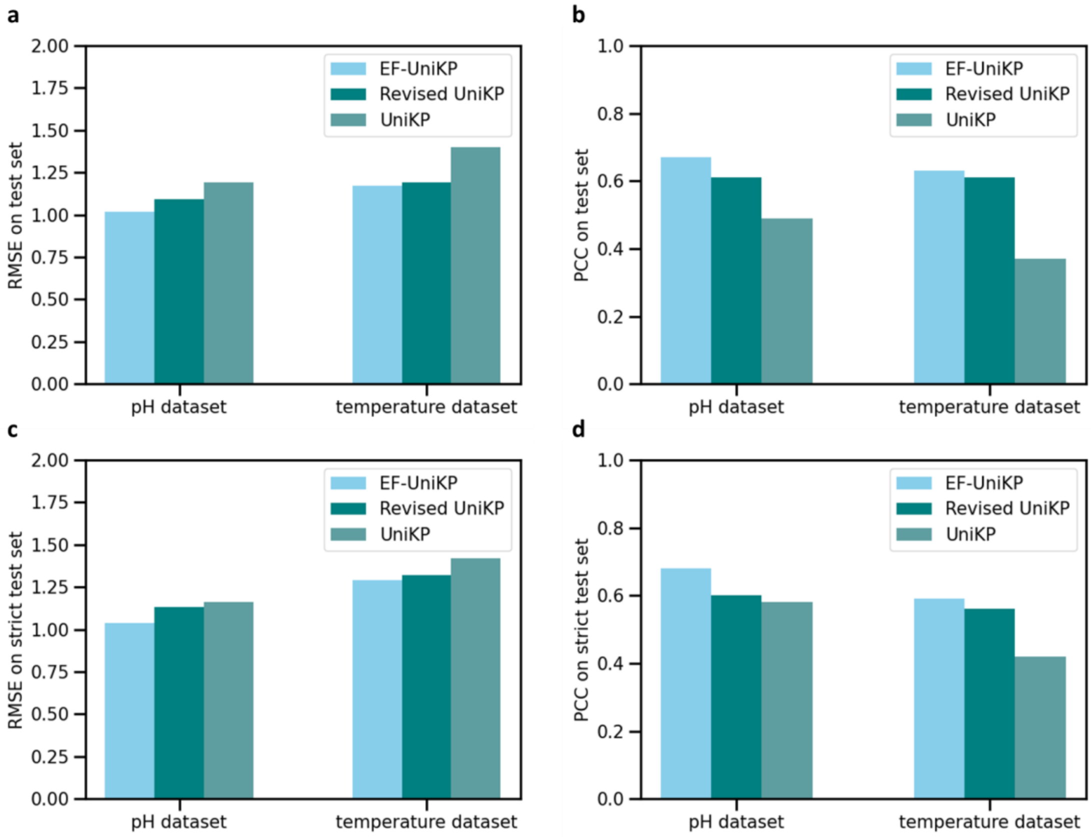

Supplementary Figure 6. a,b) Root mean square error (RMSE) (**a**) and Pearson coefficient correlation (PCC) (**b**) values between experimentally measured k_{cat} values and predicted k_{cat} values on pH and temperature test sets of EF-UniKP, Revised UniKP and UniKP. Light bars represent values of EF-UniKP, dark bars for Revised UniKP and darkish bars for UniKP. **c,d)** RMSE (**c**) and PCC (**d**) values between experimentally measured k_{cat} values and predicted k_{cat} values on more strict pH and temperature test sets of EF-UniKP, Revised UniKP and UniKP. Light bars represent values of EF-UniKP, dark bars for Revised UniKP and darkish bars for UniKP (N=636 for pH test set, N=114 for temperature test set). Source data are provided as a Source Data file.

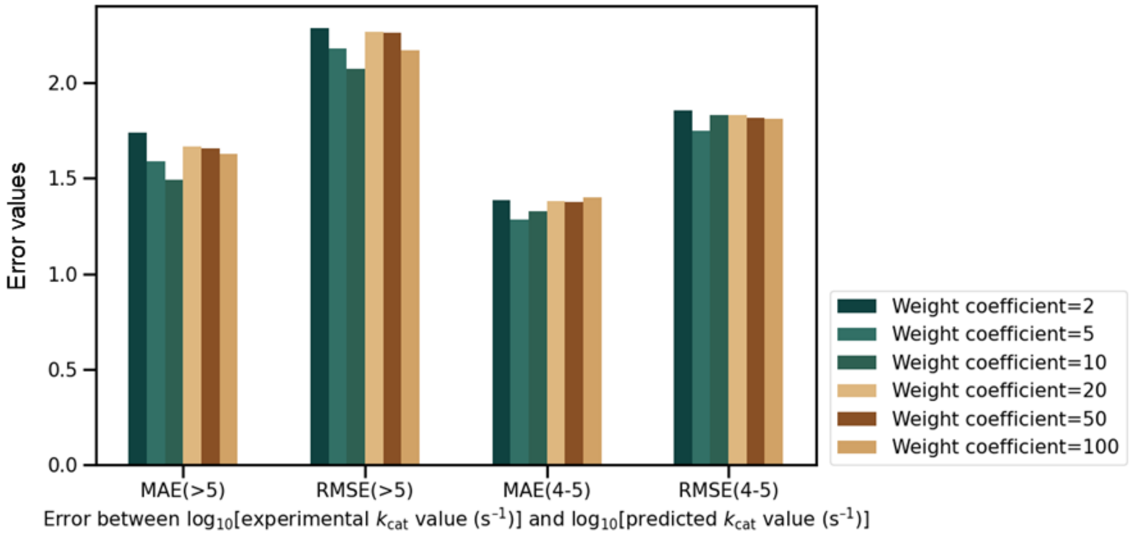

Supplementary Figure 7. Prediction errors (Mean absolute error (MAE) and Root mean square error (RMSE)) of Directly Modified Sample Weight (DMW) method with different parameters in k_{cat} intervals between 4-5 (N=90) and above 5 (>5) (N=59). Source data are provided as a Source Data file.

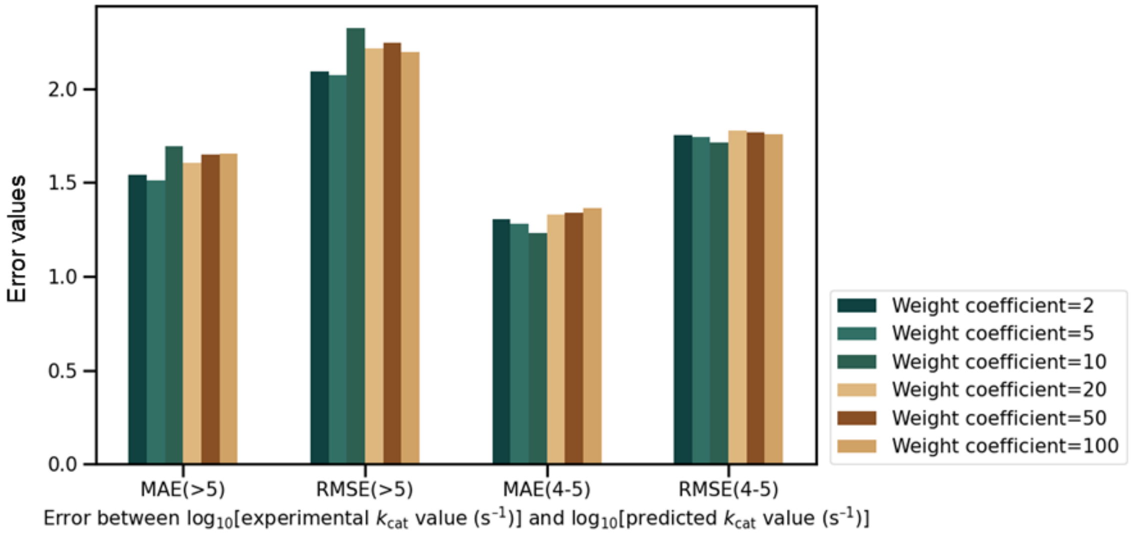

Supplementary Figure 8. Prediction errors (Mean absolute error (MAE) and Root mean square error (RMSE)) of Normalized Directly Modified Sample Weight (DMW_N) method with different parameters in k_{cat} intervals between 4-5 (N=90) and above 5 (>5) (N=59). Source data are provided as a Source Data file.

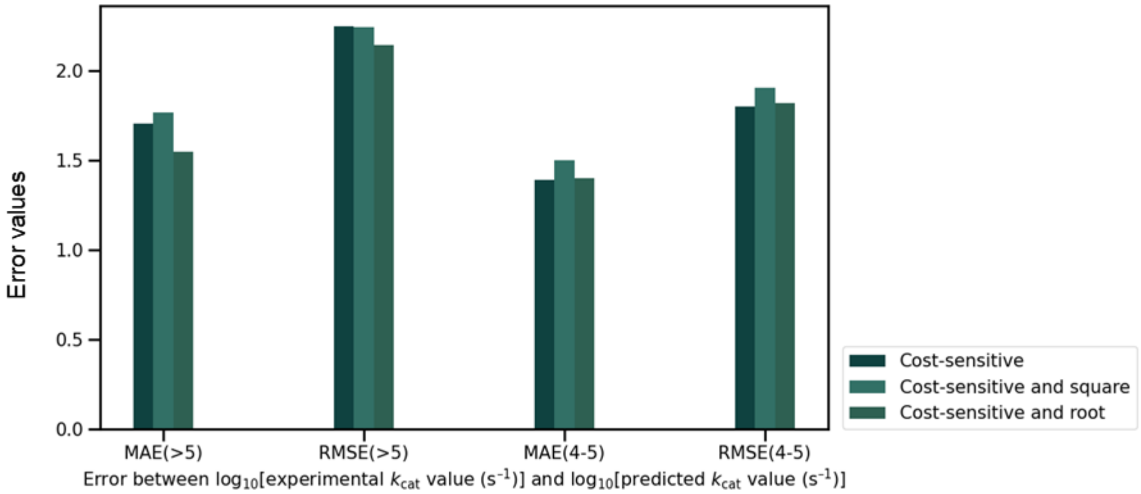

Supplementary Figure 9. Prediction errors (Mean absolute error (MAE) and Root mean square error (RMSE)) of Cost-Sensitive re-Weighting (CSW) method with different parameters in k_{cat} intervals between 4-5 (N=90) and above 5 (>5) (N=59). Source data are provided as a Source Data file.

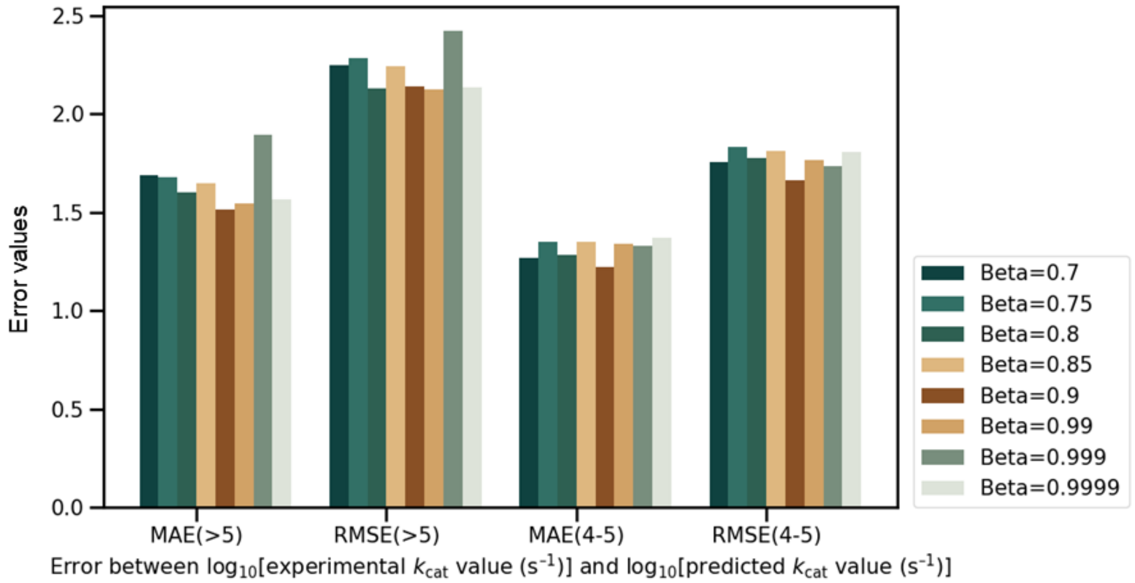

Supplementary Figure 10. Prediction errors (Mean absolute error (MAE) and Root mean square error (RMSE)) of Class-Balanced re-Weighting (CBW) method with different parameters in k_{cat} intervals between 4-5 (N=90) and above 5 (>5) (N=59). Source data are provided as a Source Data file.

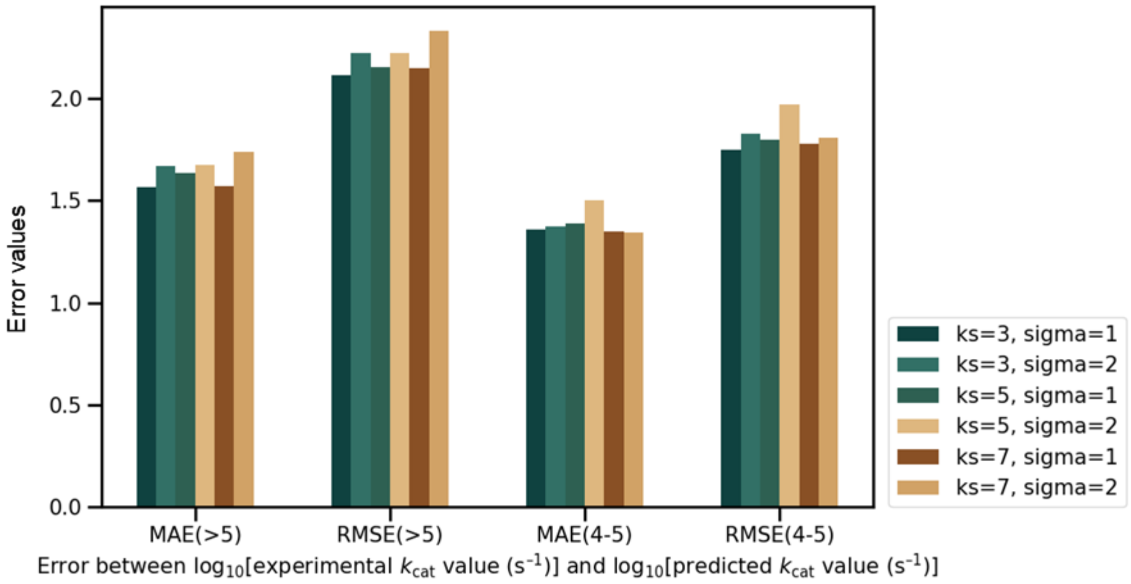

Supplementary Figure 11. Prediction errors (Mean absolute error (MAE) and Root mean square error (RMSE)) of Label Distribution Smoothing (LDS) method with different parameters in k_{cat} intervals between 4-5 (N=90) and above 5 (>5) (N=59). Source data are provided as a Source Data file.

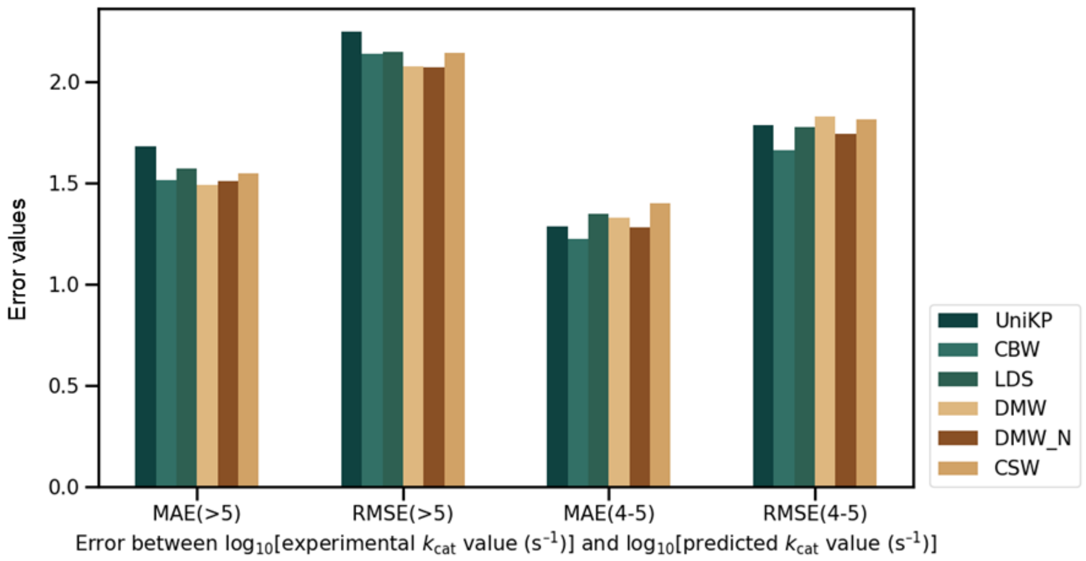

Supplementary Figure 12. Prediction Errors (Mean absolute error (MAE) and Root mean square error (RMSE)) between the predicted and experimentally measured k_{cat} values in k_{cat} intervals between 4-5 (N=90) and above 5 (>5) (N=59) using various re-weighting methods and the initial UniKP model. These representative weight redistribution methods included Class-Balanced re-Weighting methods (CBW), and Label Distribution Smoothing (LDS), Directly Modified Sample Weight (DMW), Normalized Directly Modified Sample Weight (DMW_N), Cost-Sensitive re-Weighting methods (CSW). Source data are provided as a Source Data file.

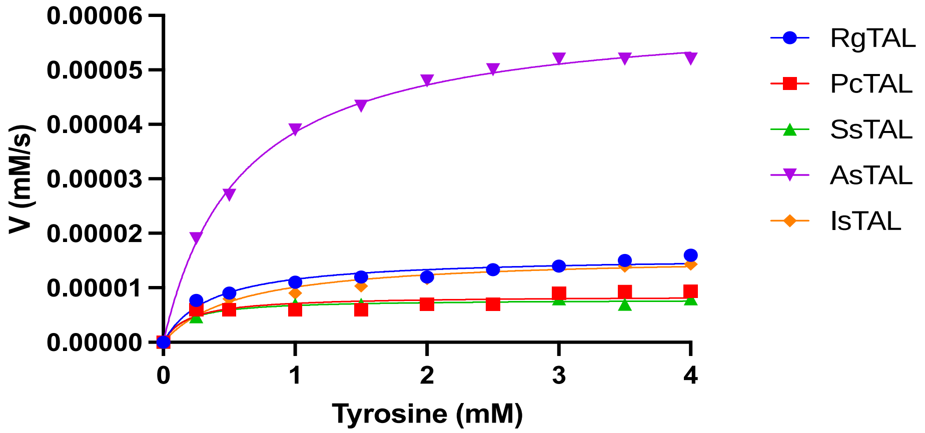

Supplementary Figure 13. The kinetic curves of wild-type Tyrosine ammonia lyase (RgTAL) from *Rhodotorula glutinis* and newly discovered TALs mined from non-redundant protein database by performing BLASTp. The 4 of top 5 sequences with the highest predicted k_{cat} values by UniKP were selected for experimental validation, including PcTAL from *Puccinia coronata f. sp. avenae* (PLW06342.1), SsTAL from *Sporidiobolus salmonicolor* (CEQ38810.1), AsTAL from *Armillaria solidipes* (BK74450.1), IsTAL from *Ilyonectria sp. MPI-CAGE-AT-0026* (KAH6995648.1). Source data are provided as a Source Data file.

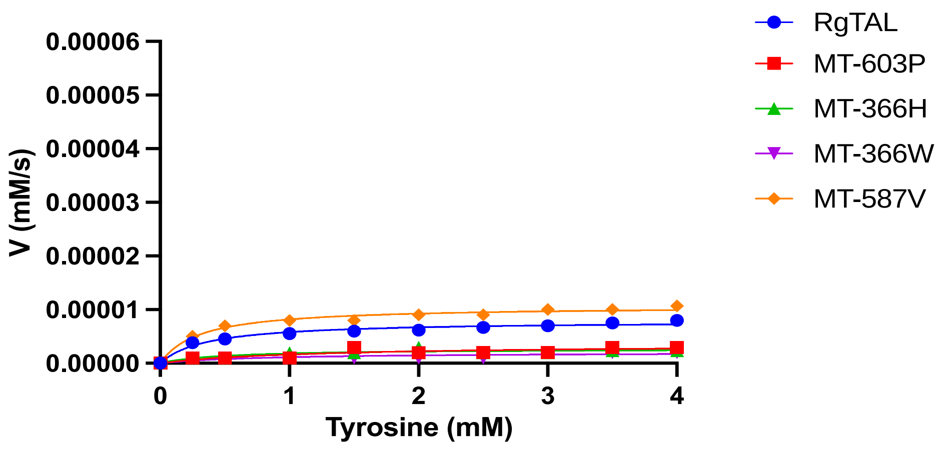

Supplementary Figure 14. The kinetic curves of Tyrosine ammonia lyase (RgTAL) from *Rhodotorula glutinis* and mutants generated by UniKP. All variants of single-point mutations were generated for RgTAL, where each variant involved mutating an amino acid at a specific position to one of the other 19 canonical amino acids, which resulted in a total number of variants equal to the product of 19 and the length of the sequence (19*693=13,167). Through an in-silico screening of all 13,167 single-point mutations of RgTAL using UniKP, the 4 of top 5 mutants ranked by their predicted k_{cat} values were chosen from each screening (k_{cat}) for experimental validation. MT denotes the mutated form of RgTAL. Source data are provided as a Source Data file.

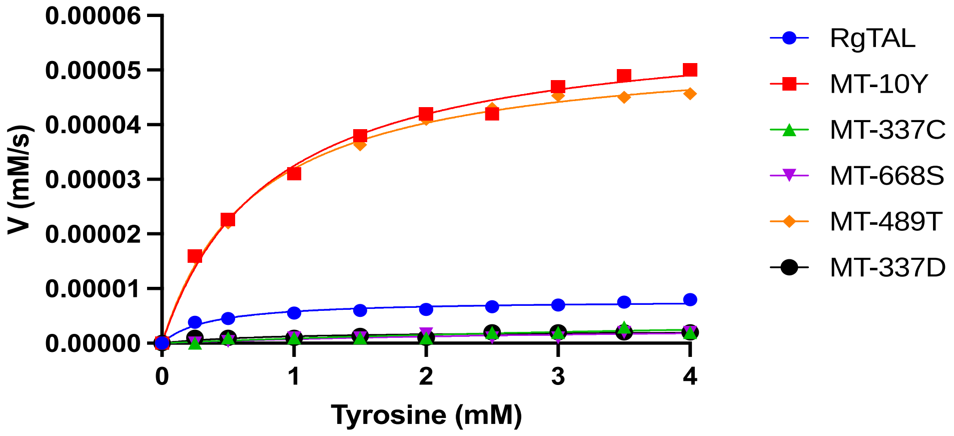

Supplementary Figure 15. The kinetic curves of Tyrosine ammonia lyase (RgTAL) from *Rhodotorula glutinis* and mutants generated by UniKP. All variants of single-point mutations were generated for RgTAL, where each variant involved mutating an amino acid at a specific position to one of the other 19 canonical amino acids, which resulted in a total number of variants equal to the product of 19 and the length of the sequence (19*693=13,167). Through an in-silico screening of all 13,167 single-point mutations of RgTAL using UniKP, the top 5 mutants ranked by their predicted k_{cat} / K_m values were chosen from each screening (k_{cat} / K_m) for experimental validation. MT denotes the mutated form of RgTAL. Source data are provided as a Source Data file.

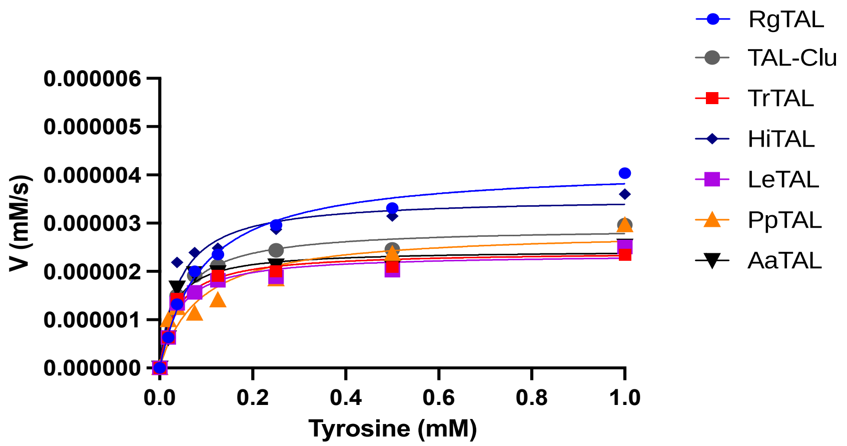

Supplementary Figure 16. The kinetic curves of wild-type Tyrosine ammonia lyase from *Rhodotorula glutinis* and *Chryseobacterium luteum* sp. nov (RgTAL, TALclu) and newly discovered TALs mined from non-redundant protein database by performing BLASTp. The top 5 sequences with the highest predicted k_{cat} values by UniKP were selected for experimental validation, including TrTAL from *Tephrocybe rancida* (KAG6920185.1), HiTAL from *Heterobasidion irregulare* TC 32-1 (XP_009553370.1), LeTAL from *Lentinula edodes* (KAF8828722.1), PpTAL from *Pleurotus pulmonarius* (KAF4563271.1), AaTAL from *Aspergillus arachidicola* (KAE8337485.1). All the experiments were conducted under a pH of 9.5. Source data are provided as a Source Data file.

Supplementary Table 1. Strains used in this study.

Strains	Relevant information	Sources
DH5α	F-ф80dlacZ△M15△ (*lacZYAIargF*)*U169deoRrecA1endA1hsdR17*(r_K^- m_K^+) *phoA*△ *supE44thi-1gyrA96 relA1*	New England Biolabs
BL21(DE3)	F^- *ompThsdS*$_B$ (rB^-mB^-) *galdcm* (DE3)	Invitrogen

Supplementary Table 2. Plasmids used in this study.

Plasmids	Relevant information	Notes
pET32a	Amp^R, pBR322origin of replication	Novagen
pET32a-RgTAL	Amp^R, pBR322origin of replication	Lab stock
pET32a-HiTAL	Amp^R, pBR322origin of replication	This study
pET32a-PcTAL	Amp^R, pBR322origin of replication	This study
pET32a-SsTAL	Amp^R, pBR322origin of replication	This study
pET32a-AsTAL	Amp^R, pBR322origin of replication	This study
pET32a-IsTAL	Amp^R, pBR322origin of replication	This study
pET32a-LiTAL	Amp^R, pBR322origin of replication	This study
pET32a-TaTAL	Amp^R, pBR322origin of replication	This study
pET32a-TALclu	Amp^R, pBR322origin of replication	This study
pET32a-TrTAL	Amp^R, pBR322origin of replication	This study
pET32a-LeTAL	Amp^R, pBR322origin of replication	This study
pET32a-PpTAL	Amp^R, pBR322origin of replication	This study
pET32a-AaTAL	Amp^R, pBR322origin of replication	This study
