# Supplementary material for: UniKP: a unified framework for the prediction of enzyme kinetic parameters
Source: Nat Commun. 2023 Dec 11;14:8211. doi: 10.1038/s41467-023-44113-1 (PMC10713628; doi:10.1038/s41467-023-44113-1)
Supplement: Supplementary file 3 — Reporting Summary [file 41467_2023_44113_MOESM3_ESM.pdf]

nature portfolio

Corresponding author(s): Xiaozhou Luo

Last updated by author(s): Nov 15, 2023

Reporting Summary

Nature Portfolio wishes to improve the reproducibility of the work that we publish. This form provides structure for consistency and transparency in reporting. For further information on Nature Portfolio policies, see our Editorial Policies and the Editorial Policy Checklist.

Statistics

For all statistical analyses, confirm that the following items are present in the figure legend, table legend, main text, or Methods section.

n/a	Confirmed	
☐	☒	The exact sample size (*n*) for each experimental group/condition, given as a discrete number and unit of measurement
☐	☒	A statement on whether measurements were taken from distinct samples or whether the same sample was measured repeatedly
☐	☒	The statistical test(s) used AND whether they are one- or two-sided<br>*Only common tests should be described solely by name; describe more complex techniques in the Methods section.*
☒	☐	A description of all covariates tested
☒	☐	A description of any assumptions or corrections, such as tests of normality and adjustment for multiple comparisons
☐	☒	A full description of the statistical parameters including central tendency (e.g. means) or other basic estimates (e.g. regression coefficient) AND variation (e.g. standard deviation) or associated estimates of uncertainty (e.g. confidence intervals)
☐	☒	For null hypothesis testing, the test statistic (e.g. F, t, r) with confidence intervals, effect sizes, degrees of freedom and P value noted<br>*Give P values as exact values whenever suitable.*
☒	☐	For Bayesian analysis, information on the choice of priors and Markov chain Monte Carlo settings
☒	☐	For hierarchical and complex designs, identification of the appropriate level for tests and full reporting of outcomes
☐	☒	Estimates of effect sizes (e.g. Cohen's *d*, Pearson's *r*), indicating how they were calculated

Our web collection on statistics for biologists contains articles on many of the points above.

Software and code

Policy information about availability of computer code

Data collection	Custom code used in this study is publicly available at https://github.com/Luo-SynBioLab/UniKP.
Data analysis	Custom code used in this study is publicly available at https://github.com/Luo-SynBioLab/UniKP.

For manuscripts utilizing custom algorithms or software that are central to the research but not yet described in published literature, software must be made available to editors and reviewers. We strongly encourage code deposition in a community repository (e.g. GitHub). See the Nature Portfolio guidelines for submitting code & software for further information.

Data

Policy information about availability of data

All manuscripts must include a data availability statement. This statement should provide the following information, where applicable:

- Accession codes, unique identifiers, or web links for publicly available datasets
- A description of any restrictions on data availability
- For clinical datasets or third party data, please ensure that the statement adheres to our policy

All the data analyzed in this study is publicly available from either public databases, including BRENDA (https://www.brenda-enzymes.org/), UniProt (https://www.uniprot.org/), PubChem (https://www.uniprot.org/) databases or supplementary datasets of referenced articles (https://github.com/SysBioChalmers/DLKcat, https://github.com/AlexanderKroll/KM_prediction). The data described in this manuscript are available for download at https://github.com/Luo-SynBioLab/UniKP. The code and data are also available as a supplementary dataset on Zenodo: https://doi.org/10.5281/zenodo.10115498.

Research involving human participants, their data, or biological material

Policy information about studies with human participants or human data. See also policy information about sex, gender (identity/presentation), and sexual orientation and race, ethnicity and racism.

Reporting on sex and gender	Not applicable in this study.
Reporting on race, ethnicity, or other socially relevant groupings	Not applicable in this study.
Population characteristics	Not applicable in this study.
Recruitment	Not applicable in this study.
Ethics oversight	Not applicable in this study.

Note that full information on the approval of the study protocol must also be provided in the manuscript.

Field-specific reporting

Please select the one below that is the best fit for your research. If you are not sure, read the appropriate sections before making your selection.

☒ Life sciences ☐ Behavioural & social sciences ☐ Ecological, evolutionary & environmental sciences

For a reference copy of the document with all sections, see nature.com/documents/nr-reporting-summary-flat.pdf

Life sciences study design

All studies must disclose on these points even when the disclosure is negative.

Sample size	These datasets were randomly split into training and test sets, 80% and 20%, respectively. The performance was evaluated directly from the test set without any assumption of underlying distributions. The number of samples is large enough to train a machine learning model, as evidenced by its excellent performance on an independent test set and the DLKcat instruction.
Data exclusions	We excluded samples with substrate simplified molecular-input line-entry system (SMILES) containing "." or kcat values less than or equal to 0, as per the DLKcat instruction.
Replication	The model parameter is available as standalone software to run on a local computer for results replication.
Randomization	All datasets were randomly split into training and test sets to ensure a fair comparison. And based on the goal of enzyme evolution, samples with the highest enzyme kinetic parameters predicted by UniKP were selected for experimental validation.
Blinding	For a fair comparison, the test sets used for UniKP performance measures were completely independent of data used for model training. And we made a comparison based on different sequence similarity. We were not blinded to allocation during experiments and outcome assessment.

Reporting for specific materials, systems and methods

We require information from authors about some types of materials, experimental systems and methods used in many studies. Here, indicate whether each material, system or method listed is relevant to your study. If you are not sure if a list item applies to your research, read the appropriate section before selecting a response.

Materials & experimental systems

n/a	Involved in the study	
☒	☐	Antibodies
☒	☐	Eukaryotic cell lines
☒	☐	Palaeontology and archaeology
☒	☐	Animals and other organisms
☒	☐	Clinical data
☒	☐	Dual use research of concern
☒	☐	Plants

Methods

n/a	Involved in the study	
☒	☐	ChIP-seq
☒	☐	Flow cytometry
☒	☐	MRI-based neuroimaging
